# Implementation and performance of a nurse administered modified PEN-FAST clinical decision rule in the electronic health record

**DOI:** 10.1017/ash.2025.10066

**Published:** 2025-07-18

**Authors:** Wesley J. Hoffmann, Shivani Patel, Elizabeth J. Lee, Natalie A. Finch, Christy P. Su, Nicole A. Teran, Yao-Hsuan Huang, Fadi Shehadeh, Muhammad Yasser Alsafadi

**Affiliations:** 1 Department of Pharmacy, Houston Methodist Hospital, Houston, TX, USA; 2 Department of Pharmacy, Houston Methodist The Woodlands, Conroe, TX, USA; 3 Department of Pharmacy, Houston Methodist West, Katy, TX, USA; 4 Department of Pharmacy, Houston Methodist Willowbrook, Houston, TX, USA; 5 Department of Medicine, Houston Methodist Hospital, Houston, TX, USA; 6 Department of Electrical Engineering and Computer Engineering, National Technical University of Athens, Athens, Greece; 7 Division of Infectious Diseases, Department of Medicine, Houston Methodist Hospital, Houston, TX, USA

## Abstract

**Objective:**

To evaluate performance of registered nurse assessments of the PEN-FAST penicillin allergy clinical decision rule compared to antimicrobial stewardship pharmacists.

**Design:**

Prospective, blinded, non-interventional, quality assurance study.

**Setting:**

This study took place across 4 inpatient hospitals within a large health system in Houston, Texas.

**Methods:**

We implemented PEN-FAST rule questions into the electronic health record (EHR) for registered nurses to perform. Patients were randomly selected in a prospective fashion, with nurse documented scores hidden, for re-assessment by antimicrobial stewardship pharmacists to compare risk stratification and scores.

**Results:**

Overall agreement of high risk and low risk results was 84.3%. Registered nurse evaluations with the PEN-FAST clinical decision rule for detecting a high-risk patient demonstrated a sensitivity of 67%, specificity of 89.8%, positive predictive value of 67.9%, and negative predictive value of 89.5%. Additionally, 34.4% of patients with a documented penicillin allergy admitted to tolerating amoxicillin or amoxicillin/clavulanate since their last recalled reaction to penicillin.

**Conclusions:**

Registered nurse assessment of the PEN-FAST clinical decision rule demonstrated good performance and can effectively be used to screen for low-risk penicillin allergy patients. Incorporation of the PEN-FAST rule into EHR can be scaled into large health systems to help appropriately stratify patients with low- and high-risk penicillin allergies and improve documentation.

## Introduction

Penicillin allergies are a significant concern in healthcare, with about 10% of the U.S. population reporting such an allergy. However, 90–95% of these self-reported penicillin allergies are inaccurate.^1-6^ Additionally, penicillin allergies can diminish over time, with about 50% of people losing their allergies within five years and 80% within ten years.^5,7^ Misreporting penicillin allergies has serious consequences, often leading to unnecessary use of broad-spectrum antibiotics. Moreover, non-beta-lactam antibiotics are more likely to cause adverse effects and increase the risk of resistant infections like methicillin-resistant *Staphylococcus aureus* and *Clostridioides difficile*.^2,8^ Given these risks, it is crucial to verify and reassess penicillin allergies to ensure patients receive the most effective antibiotic therapy.^9,10^

Several approaches are available for penicillin allergy assessments. Penicillin skin testing is one reliable method to detect IgE-mediated hypersensitivity.^7^ However, skin testing is resource-intensive, includes 1–2 hours of clinician time and should be performed by a healthcare professional trained in allergy testing. Moreover, the shortage of allergists in the U.S. exacerbates the issue, with 81.5% of U.S. counties lacking an allergist and only 1.71 allergists per 100,000 people.^11^ A direct oral challenge is another method to consider and is more practical than skin testing for patients with low risk of significant reactions. This typically includes the administration of a 250 mg dose of amoxicillin with close monitoring for 60 minutes. A validated clinical decision rule called PEN-FAST can be utilized to assess penicillin allergy risk at the point of care.^12^ This clinical decision rule identifies patients with a lower risk (PEN-FAST score <3) that do not need formal skin testing, streamlining penicillin allergy assessment and management. A recent randomized clinical trial, the Penicillin Allergy Clinical Decision Rule (PALACE), demonstrated that patients with low risk can safely receive a direct oral penicillin challenge.^13^ The trial reported a very low incidence (0.5%) of immune-mediated reactions in both the direct challenge and the control group, which underwent penicillin skin testing followed by an oral challenge.

Building on the success of the PEN-FAST clinical decision rule and the PALACE trial, as well as nurse driven allergy assessments demonstrating success in inpatient settings,^14^ we implemented a nurse-driven modified PEN-FAST clinical decision rule for all patients admitted to the acute care setting across our eight-hospital healthcare system. Next, we evaluated the consistency of PEN-FAST assessments between registered nurses (hereafter referred to as nurses or nursing) and seven antimicrobial stewardship (AMS) pharmacists (referred to as AMS pharmacists). Six of these AMS pharmacists had completed a residency in infectious diseases clinical pharmacy and the seventh had completed a residency in critical care pharmacy and had several years of dedicated experience in antimicrobial stewardship at a community hospital. This provides a framework for standardizing and operationalizing penicillin allergy assessments through nursing collaboration and electronic health record (EHR) integration.

## Methods

### Study design

We conducted a prospective, observational, non-interventional, quality assurance study of patients who received a nurse-driven penicillin allergy assessment, modified from the PEN-FAST clinical decision rule. A random subset of patients was selected for a second assessment performed by AMS pharmacists. This study was performed on admission or at the earliest practical opportunity thereafter across four hospitals within the Houston Methodist (HM) Health System in Houston, Texas.

### PEN-FAST questionnaire modifications

**Figure 1.**
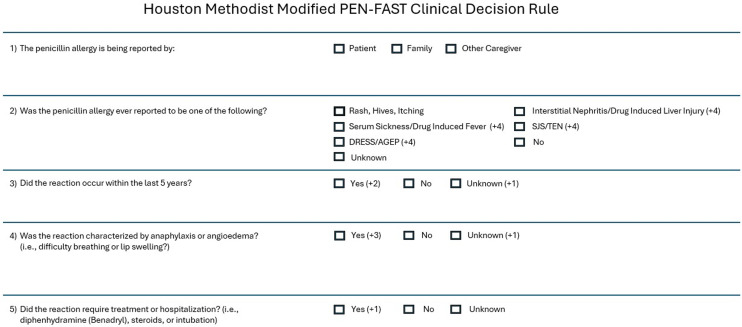
Houston methodist modified PEN-FAST clinical decision rule.

A modified version of the PEN-FAST questionnaire was incorporated into the admission navigator of the EHR for use by nursing staff or clinicians (Figure 1). As a safety measure, the PEN-FAST questionnaire was modified to ensure that no patients with a reported history of severe IgE mediated reactions could be miscategorized as low risk. Key modifications include increasing the score for anaphylaxis or angioedema to 3 points (from 2 points). Additionally, a score of 1 point was assigned for “unknown” responses to the questions: “Did the reaction happen in the past 5 years?” and “Was the reaction characterized as anaphylaxis or angioedema?”. To further ensure patient safety, individuals reporting severe non-IgE mediated reactions, such as Stevens-Johnson Syndrome/Toxic Epidermal Necrolysis, Serum Sickness, Drug Rash with Eosinophilia and Systemic Symptoms, and Acute Generalized Exanthematous Pustulosis were assigned a score of 4, consistent with the recommendation that patients who exhibit these reactions should not be challenged without an allergist’s supervision.

### Best practice alert integration and scoring

As part of an antimicrobial stewardship initiative focused on penicillin allergy assessment and management (and approved by Houston Methodist system clinical nursing committee), a best-practice alert was triggered in the Epic electronic health record (EHR) system for patients with a penicillin class allergy label. This alert and the questionnaire were custom built into Epic locally for purposes of hospital antimicrobial stewardship and to enhance allergy documentation. The alert prompted registered nurses to complete the penicillin assessment questionnaire within the admission navigator for patients admitted to the hospital, in observation status, the emergency department, or for same-day surgery. Each selected response on the questionnaire was assigned a point value, with modifications to the original PEN-FAST clinical decision rule as detailed above. The final score was auto-calculated based on the responses and saved in the patient’s electronic health record under the allergies tab, making it accessible to all healthcare providers. Education regarding the data surrounding penicillin allergy, the scoring tool, and its integration into Epic was developed in conjunction with Nursing Education and provided as a required stand-alone training module with a competency-based assessment for nursing and pharmacy staff across the system via the Houston Methodist learning management system.

### Data management

The Houston Methodist Institutional Review Board reviewed the study protocol and granted it exempt status. Informed consent was waived, as this was part of a quality assurance project, and all patient data were de-identified. Data was de-identified and provided a patient link to avoid any PHI breaches and stored in secured, password protected files.

### Patient identification and reporting

A report of all admitted patients with a documented allergy to penicillin class and a completed penicillin assessment score was generated through Epic reports at least three times per week (on Mondays, Wednesdays, and Fridays) or more frequently based on clinician availability. To identify patients appropriate for evaluation, the report (which listed all patients with a penicillin allergy AND a PEN-FAST score within the Houston Methodist (HM) Health System) was filtered to include only those who were admitted or in observation status at one of the facilities participating in the evaluation (HM Hospital—Texas Medical Center (TMC), HM West, HM The Woodlands, and HM Willowbrook).

### Concealment

The curated list was prepared and distributed by a member of the antimicrobial stewardship team from HMH-TMC (author WJH) and securely sent to the AMS pharmacist at each respective hospital. Each patient’s penicillin assessment score was concealed, and the list was randomized to avoid targeting specific scores before being sent to assessors. The only information provided to the AMS pharmacists was the patient’s name, date of birth, admission date, and location within the hospital.

### Penicillin allergy assessments

Assessors interviewed and performed penicillin allergy assessments using the same questionnaire available in Epic for patients on the list prior to accessing their charts, deliberately avoiding any visibility of the penicillin assessment scores to prevent potential bias.

As part of the AMS pharmacist interview, at the end of the questionnaire, patients were asked if they had ever tolerated amoxicillin or amoxicillin/clavulanate (U.S. brand name Augmentin) without issue any time after their reported penicillin allergy reaction. AMS pharmacists at each institution interviewed as many patients as possible during their normal workday, documented answers to each question in a secure external data collection tool outside of the EHR, and recorded final scores based on their own assessment. After completing their assessments (which were then locked in, so answers could not be changed), the AMS pharmacists reviewed and transcribed the nursing penicillin assessment scores for each patient into the data collection file. Scores were recorded under unique identifiers for each patient and kept in a separate file to ensure patient de-identification.

### Inclusion and exclusion criteria

Patients were included in the study if both nursing and AMS pharmacist assessments were completed. Exclusion criteria applied to patients whose nursing assessments were completed before July 1, 2024 (the first weekday the finalized questionnaire was available). Patients were excluded if they were discharged before reassessment by a member of the antimicrobial stewardship team, if they were absent from their room during the visit, if they were in surgery or the PACU at the time of assessment and not subsequently admitted, or if they refused a repeat assessment or were unable to communicate effectively as determined by the clinicians. Patients were also excluded if the nurse’s assessment was incomplete (ie, not all questions were answered) or if the assessment was performed but the patient did not have a listed penicillin allergy. Patients with multiple assessments by pharmacy only had their first assessment included.

### Statistical analysis

All analyses were performed using Stata 18 (StataCorp, College Station, TX) and Python 3. Data in Table 1 were summarized as median (interquartile range) for continuous variables and frequency (percentage) for categorical variables. Categorical variables were compared using Pearson’s χ^2^ test, and continuous variables were compared using the Wilcoxon rank-sum test. The antimicrobial stewardship pharmacists’ evaluations served as the reference standard, while the registered nurse evaluations served as the predicted classification.

**Table 1.** Patient demographics

	Total (*n* = 338)	Low Risk Modified PEN-FAST Score (*n* = 256)	High Risk Modified PEN-FAST Score (*n* = 82)	*p*-value
Sex				.002
Female	228 (67.5%)	161 (62.9%)	67 (81.7%)	
Male	110 (32.5%)	95 (37.1%)	15 (18.3%)	
Age (in years)	68.00 (55.00 – 77.00)	70.00 (57.00 – 79.00)	60.50 (50.00 – 73.00)	.003
Race				.006
Asian	10 (3.0%)	9 (3.5%)	1 (1.2%)	
Hispanic or Latino	42 (12.4%)	23 (9.0%)	19 (23.2%)	
Non-Hispanic Black	66 (19.5%)	47 (18.4%)	19 (23.2%)	
Non-Hispanic White	216 (63.9%)	174 (68.0%)	42 (51.2%)	
Other / Unknown	4 (1.2%)	3 (1.2%)	1 (1.2%)	
Myocardial Infarction	90 (26.6%)	69 (27.0%)	21 (25.6%)	.81
Congestive Heart Failure	138 (40.8%)	108 (42.2%)	30 (36.6%)	.37
Peripheral Vascular Disease	159 (47.0%)	125 (48.8%)	34 (41.5%)	.24
Cerebrovascular Disease	136 (40.2%)	101 (39.5%)	35 (42.7%)	.60
Dementia	34 (10.1%)	23 (9.0%)	11 (13.4%)	.25
Chronic Pulmonary Disease	177 (52.4%)	133 (52.0%)	44 (53.7%)	.79
Rheumatic Disease	50 (14.8%)	32 (12.5%)	18 (22.0%)	.036
Peptic Ulcer Disease	50 (14.8%)	34 (13.3%)	16 (19.5%)	.17
Mild Liver Disease	94 (27.8%)	72 (28.1%)	22 (26.8%)	.82
Diabetes Without Complications	42 (12.4%)	32 (12.5%)	10 (12.2%)	.94
Diabetes With Complications	124 (36.7%)	92 (35.9%)	32 (39.0%)	.61
Paraplegia and Hemiplegia	38 (11.2%)	23 (9.0%)	15 (18.3%)	.02
Renal Disease	157 (46.4%)	123 (48.0%)	34 (41.5%)	.30
Cancer	47 (13.9%)	39 (15.2%)	8 (9.8%)	.21
Moderate or Severe Liver Disease	21 (6.2%)	14 (5.5%)	7 (8.5%)	.32
Metastatic Solid Tumor	63 (18.6%)	45 (17.6%)	18 (22.0%)	.38

A confusion matrix was constructed to compare “high risk” classifications (score ≥ 3). In this setup, a true positive occurred when both pharmacist and nurse classified a patient as high risk, whereas a false positive occurred when the nurse classified a patient as high risk but the pharmacist did not. A true negative occurred when both pharmacist and nurse classified a patient as low risk, while a false negative occurred when the nurse classified a patient as low risk but the pharmacist did not.

From the confusion matrix, sensitivity (the number of true positives divided by the sum of true positives and false negatives), specificity (the number of true negatives divided by the sum of true negatives and false positives), positive predictive value (the number of true positives divided by the sum of true positives and false positives), negative predictive value (the number of true negatives divided by the sum of true negatives and false negatives), and accuracy (the sum of true positives and true negatives divided by the total number of assessments) were calculated.

## Results

Patients were enrolled between July 2024 and November 2024. The cohort included 338 patients, comprising 110 male (32.5%) and 228 female (67.5%). Patient demographics are shown in Table 1. Patient response rates to the PEN-FAST clinical decision rule, as assessed by nurses and AMS pharmacists, are presented in Table 2.

**Table 2.** Modified PEN-FAST responses

	RN Group (*n* = 338)	AMS Pharmacist Group (*n* = 338)	Significance
Low-risk category (score <3)	257 (76.5%)	256 (76.2%)	
High-risk category (score ≥3)	81 (23.5%)	82 (23.8%)	
Question 1—The penicillin allergy is being reported by*:			*P* = .29
Patient	304 (89.9%)	311 (92.0%)	
Family	32 (9.5%)	27 (8.0%)	
Question 2—Was the reaction ever reported to be one of the following?†			*P* = .45
Rash/itching/hives	211 (62.4%)	221 (65.4%)	
‘Unknown’ or ‘none of the above’	122 (36.1%)	116 (34.3%)	
Question 3—Did the reaction occur in the last 5 years?			*P* < .001
No	259 (76.6%)	301 (89.1%)	
Yes	40 (11.8%)	32 (9.5%)	
Unknown	39 (11.5%)	5 (1.5%)	
Question 4—Was the reaction characterized as anaphylaxis or angioedema?			*P* = .012
Yes	65 (19.2%)	69 (20.4%)	
No	201 (59.5%)	226 (66.9%)	
Unknown	72 (21.3%)	43 (12.7%)	
Question 5—Did the reaction require treatment?			*P* = .003
Yes	84 (24.9%)	119 (35.2%)	
No	178 (52.7%)	136 (40.2%)	
Unknown	76 (22.5%)	83 (24.6%)	
Amoxicillin tolerance subsequent to initial reaction‡			
Has tolerated amoxicillin or Augmentin®	N/A	116 (33.7%)	
Unsure if they had taken amoxicillin or Augmentin®	N/A	69 (20.1%)	
Has not taken amoxicillin or Augmentin®	N/A	159 (46.2%)	
Modified PEN-FAST Score			
Average PEN-FAST Score	1 (0 – 2)	1 (0 – 2)	*P* = .36
High Risk ( ≥ 3)	81 (24.0%)	82 (24.3%)	*P* = .93
Low Risk ( <3)	257 (76.0%)	256 (75.7%)	*P* = .93
Modified PEN-FAST Score Breakdown			
Score: 0	147 (42.7%)	154 (44.7%)	*P* = .59
Score: 1	68 (19.7%)	86 (25%)	*P* = .10
Score: 2	48 (14%)	42 (12.2%)	*P* = .50
Score: 3	25 (7.3%)	29 (8.4%)	*P* = .57
Score: 4+	56 (16.3%)	53 (15.4%)	*P* = .75

* 2 patients in the nursing group were reported as ‘unknown’.

†In the nursing group, the remaining 5 patients were reported as follows: DRESS (2), Serum Sickness (2), SJS (1). In the ID PharmD group, 1 patient reported DRESS.

‡This question was only part of the ID PharmD Questionnaire.

Overall, nursing assessments demonstrated an accuracy of 84.3% compared to AMS pharmacist evaluations, with concordance on low-risk status in 230 patients (68%) and high-risk status in 55 patients (16.3%). Among discordant cases, nurses assigned higher scores, leading to false high-risk scores in 26 patients (7.7%), and lower scores, resulting in false low-risk scores for 27 patients (7.9%). The penicillin allergy assessment conducted by registered nurses showed a sensitivity of 67%, specificity of 89.8%, positive predictive value (PPV) of 67.9%, and a negative predictive value (NPV) of 89.5% for identifying high-risk allergies (Table 3).

**Table 3.** Modified PEN-FAST clinical decision rule accuracy

Metric	Percentage
Sensitivity	67%
Specificity	89.8%
Positive Predictive Value	67.9%
Negative Predictive Value	89.5%

When identifying anaphylaxis/angioedema, the nursing assessment exhibited sensitivity, specificity, PPV, and NPV of 63.8%, 92.0%, 71%, and 89.3%, respectively. For the question “Was the reaction ever characterized as anaphylaxis/angioedema?”, a patient response of “unknown” (rather than “yes” or “no”) was documented in 72 (21.3%) cases by nursing staff. After assessment and further inquiry by the AMS pharmacist regarding symptoms, patients clarified an answer of “yes” to anaphylaxis/angioedema in 8 of 72 (11.1%) of these patients; 44 patients (61.1%) answered “no” while the remaining 20 (27.8%) continued to respond as “unknown.”

The impact of our modified clinical decision rule for the anaphylaxis/angioedema (+3 points vs +2 points in PEN-FAST) question on risk stratification was also analyzed. Patients were classified into the high-risk category based solely on a “yes” response to this question in 12 patients (3.5%) in the nursing group compared to 16 (4.7%) in the AMS pharmacist group. Of the 25 patients whom nursing staff documented as “no” or “unknown” but the AMS pharmacist group had classified as anaphylaxis/angioedema, 4 (16%) patients were still categorized as high-risk due to points accrued from other questions.

A few trends were observed when comparing nurse and AMS pharmacist assessments. Nurses were more likely to document “unknown” for the question regarding whether the reaction occurred in the last 5 years or not (11.5% vs 1.5%), whereas AMS pharmacists were more likely to answer “no” (89.1% vs 76.6%). In addition, nursing staff were more likely to answer “unknown” (21.3% vs 12.7%) and AMS pharmacists were more likely to answer “no” (66.9% vs 59.5%) to the question, “Was the reaction characterized by anaphylaxis or angioedema?”. Lastly, nurses were also more likely to document that patients did not require treatment compared to the AMS pharmacists (52.7% vs 40.2%).

Additionally, the AMS pharmacist cohort incorporated an extra interview question to determine whether patients had ever tolerated “amoxicillin” or “amoxicillin/clavulanate (Augmentin®)” *after* their initial reaction to penicillin class agents. Results showed that 116 patients (34.3%) reported tolerating amoxicillin and/or amoxicillin/clavulanate after their initial reaction, while 67 patients (19.8%) were unsure (ie, the drug names sounded familiar but could not confirm prior use). The remaining 155 patients (46.2%) had not taken amoxicillin or amoxicillin/clavulanate again.

Furthermore, among the 69 patients in the AMS pharmacist group, who reported a history of anaphylaxis, 15 (21%) acknowledged tolerating amoxicillin or amoxicillin/clavulanate since their initial reaction. The findings suggest that a significant proportion of patients who reported severe reactions may still tolerate penicillins, potentially carrying an erroneous allergy label.

## Discussion

Our study demonstrated that nursing staff are well-equipped to perform a standardized penicillin allergy assessment within the electronic medical record, using a modified PEN-FAST rule, for patients in hospital, emergency department, or surgical care settings. At our institution, registered nurses conduct a medication allergy assessment for all patients as part of their routine hospital admission workflow. Previous literature has shown that agreement between a patient’s reported reaction and the documented chart entry is 50% or lower.^15,16^ Standardized questions, such as those in PEN-FAST, provide a more accurate assessment of allergic reactions and associated risks compared to open-ended patient reporting.

Our findings illustrate that integrating a modified PEN-FAST score into nurses’ routine hospital admission workflow achieved an accuracy of nearly 85% and a high negative predictive value of 89.5% for ruling out high-risk allergies. While this does not eliminate the need for clinician reassessment before antibiotic challenges, it effectively identifies low risk patients who may be candidates for interventions such as oral antibiotic challenges or de-labeling.

Despite its simplicity and favorable performance characteristics, PEN-FAST remains underutilized, as medication allergy histories are often overlooked amidst competing clinical demands. Embedding a structured allergy assessment into the electronic medical record as a required activity and ensuring its accurate use by nursing staff addresses this gap, making critical allergy information readily available at the point of prescribing. Incorporating the PEN-FAST rule into the electronic medical record and demonstrating its effective implementation by registered nurses supports its scalability across large healthcare systems, compared to previous studies conducted primarily in smaller settings such as outpatient clinics.

While this study demonstrated a high level of agreement between nurse and AMS pharmacist PEN-FAST assessments, some limitations exist. A small but notable discordance was observed in cases where nursing staff documented a “no” response to the anaphylaxis/angioedema question, but AMS pharmacists identified symptoms of anaphylaxis or angioedema upon further inquiry. Overall, 27 of 344 patients (7.8%) fell into this false-negative category, posing a potential safety risk if PEN-FAST was utilized without independent assessment by clinicians. Additionally, 26 patients (7.6%) were erroneously classified as high risk by nursing staff while being found to be low risk by the AMS pharmacist (false positive). While false positives are less concerning from an immediate safety standpoint than false negatives, they may lead to unnecessary use of second line antibiotics, potentially impacting antimicrobial stewardship efforts. These discrepancies underscore the need for ongoing education for nursing staff to optimize assessment accuracy.

Regarding the observed error rates, several potential explanations exist. First, patients interviewed by nursing staff on the first day of their hospital admission may be in distress, affecting their ability to accurately recall their allergic reactions. In contrast, AMS pharmacists typically assess patients later, when they may be more stable and able to provide clearer responses.

Second, the admission process for registered nurses working in high acuity settings such as emergency departments, inpatient wards or same-day surgery settings involves numerous competing tasks, including urgent and life-saving responsibilities. This workload may inadvertently contribute to errors. Comparatively, AMS pharmacists who were interviewing patients had more time and experience to probe further into the types of reactions and clarify the meaning of the responses provided. Third, in these high acuity settings, the wording of questions and response options in the electronic medical record’s admission navigator may be considered lengthy or unclear by the nurse or patient, potentially leading to a misinterpretation and inaccurate response. Lastly, this study assumes that AMS pharmacists, who focus solely on allergy assessments and have specialized experience in penicillin allergy evaluation, perform more accurate assessments than balancing multiple admission-related tasks.

## Conclusion

Integrating a penicillin allergy assessment clinical decision rule into the EHR to be performed by registered nurses upon admission is an effective approach for categorizing patient allergy risk. A nurse-administered modified PEN-FAST assessment demonstrates strong concordance with assessments performed by infectious diseases pharmacists specialized in penicillin allergy management. Future research should explore how these scores influence antibiotic prescribing patterns and assess whether patients at moderate risk would benefit from targeted interventions, such as direct oral challenges and de-labeling programs.
